# Protective Effects of CVD and DM Medications in SARS-CoV-2 Infection

**DOI:** 10.1007/s42399-020-00452-4

**Published:** 2020-08-17

**Authors:** Shifa Bangi, Rajas Barve, Amna Qamar

**Affiliations:** 1 Faculty of Medicine, School of Medicine, Imperial College London, Sir Alexander Fleming Building, London, SW7 2AZ UK; 2 School of Medicine, Institute of Life Course and Medical Sciences, University of Liverpool, Liverpool, UK

**Keywords:** CVD, Diabetes, SARS-CoV-2, COVID-19, Drug repurposing

## Abstract

Despite the burden of disease of CVD and DM, there is a lack of experimentally validated literature exploring their association with exacerbation of COVID-19. Target receptors of medications commonly used to treat CVD and DM may be involved in the viral entry mechanism of SARS-CoV-2. We propose the potential protective effects of these medications in COVID-19 infections, highlighting the need for further research. Firstly, AMPK mediated phosphorylation of ACE-2 by metformin as well as the drug’s alkaline properties may interrupt the natural disease progression. Secondly, DPP4 receptor involvement in the putative viral entry of SARS-CoV-2 may be prevented by DPP4i. Finally, recent studies have shown that statins’ ability to inhibit the cytokine storm may outweigh concerns of statin mediated ACE-2 upregulation in COVID-19. The complex interplay of factors affecting CVD and DM in COVID-19 patients makes the direct effects of medications difficult to examine. Therefore, further research is needed, in the context of SARS-CoV-2 and the molecular pathways it exploits, to potentially repurpose such pre-existing drugs for their use in COVID-19.

The literature surrounding the association between cardiovascular disease (CVD) and diabetes mellitus (DM) with SARS-CoV-2 is sparse. Roy et al. propose multiple pathways to explain these complex relationships. There are two aspects pertaining to CVD; firstly, the concept that SARS-CoV-2 can propagate the development of CVD, and secondly, pre-existing CVD can exacerbate SARS-CoV-2 infections, increasing the incidence of cardiac complications. In DM, immune dysfunction increases susceptibility to severe COVID-19 infection [1]. Due to the lack of a vaccine, or targeted therapy for COVID-19, there is an urgent need for further research into potential pharmacological therapies. We propose that patients with these common comorbidities are likely on medications, which may offer protection against severe infections. This may be achieved through the drugs’ action on target receptors of SARS-CoV-2. It is crucial to identify interactions of SARS-CoV-2 with commonly used drugs, including metformin, dipeptidyl-peptidase 4 inhibitors (DPP4i) and statins, with the potential to repurpose them to prevent disease progression.

Roy et al. suggests that angiotensin-converting enzyme (ACE2) plays a key role in the severity of disease for COVID-19 patients. Its upregulation in patients with DM makes them more susceptible to complications [1]. We propose that metformin, the first-line treatment for DM, may have protective effects against the virus. Sharma et al. explain that metformin activates AMP-activated protein kinase (AMPK) leading to ACE2 phosphorylation. Theoretically, this may induce a conformational change in ACE2, making it less able to bind to SARS-CoV-2 [2]. Following infiltration of the host cells, SARS-CoV-2 downregulates ACE2 receptors leading to pro-inflammatory repercussions, via the renin-angiotensin system (RAS). As metformin activates AMPK and further downstream ACE2, it may prevent SARS-CoV-2-mediated ACE2 downregulation [2]. Additionally, Esam highlights the use of metformin in reversing lung fibrosis and provides insight into its alkaline properties. The suggestion is that the basic nature of metformin may neutralize acidic, SARS-CoV-2-containing vesicles [3]. Although the mechanisms employed by metformin may prove to be protective against severe infection, further research is required to confirm these hypotheses.

Similar to ACE2, conditions with DPP4 upregulation are associated with severe COVID-19. These include old age, obesity, chronic kidney disease (CKD) and COPD [4]. Roy et al. emphasize the role of ACE2 in viral entry [1]; however, modelling has shown that DPP4 may be a co-receptor for SARS-CoV-2 entry into respiratory tract cells via S1 spike protein interaction [4]. Gliptins, which are DPP4 inhibitors (DPP4i), are cardioprotective in CKD and have anti-inflammatory properties [4]. Contrary to Roy et al.’s suggestion that DPP4i may worsen COVID-19 progression [1], the co-receptor hypothesis suggests that DPP4i may actually have a protective effect [4]. It is possible that ACE inhibitors, which increase ACE2 expression in diabetics, may mask this protective effect in patients using the combination of ACEi and DPP4i. However, gliptins do not bind to the suggested binding site of SARS-CoV-2, meaning that the wealth of data surrounding DPP4 may serve a basis for the development of new drugs, as opposed to a direct repurposing of DPP4i for treatment of COVID-19 [4]. Since specific gliptins show different effects in rodent models, further studies are needed to identify the type of DPP4i with the greatest therapeutic potential in COVID-19. Although validation of the role of DPP4 is needed, current models suggest that cells co-expressing ACE2 and DPP4 could provide targets for antiviral therapy [4].

Since its emergence, COVID-19 has been associated with CVD. Roy et al. demonstrates the duality of this relationship as CVD is both a comorbidity and a consequence of infection [1]. Statins are widely prescribed as primary and secondary prevention in patients with high CVD risk. Research has led to a greater understanding of their mechanism of action; however, their pleiotropic effects are yet to be fully elucidated. A retrospective cohort study in China by Zhang et al., comparing 1219 patients taking statins to 12,762 control patients, showed that in-hospital use of statins is linked to reduced all-cause mortality in COVID-19 patients [5]. Despite the concerns of statins resulting in the increased expression of ACE2, this molecular response has not manifested as a clinical outcome. All-cause mortality was reduced in the statin group despite consisting of comparatively older patients with a higher number of comorbidities, including DM, hypertension and coronary heart disease [5]. This positive outcome can be partially attributed to the anti-inflammatory effects of statins, specifically their inhibitory effects on cytokines which suppress the cytokine storm present in severe COVID-19. Not only did patients in the statin group have lower levels of IL-6 on admission, they also had a smaller increase in IL-6 levels during their stay [5]. As well as preventing disease progression in CVD patients, statins may also prove beneficial in reducing the incidence of CVD complications such as myocarditis. However, there are discrepancies between observational studies and randomized controlled trials (RCTs) when investigating the effect of statins on mortality for infections [5]. Thus, further large-scale RCTs are required, within the context of COVID-19, to confirm the potential prognostic benefits of statins. It is also imperative to investigate the differences between individual statins due to the known heterogeneity in the effects within this drug class.

Widely used drugs such as metformin, DPP4i and statins may provide protection against disease progression in COVID-19 patients with common comorbidities including diabetes and CVD. The complexity of the interactions between aetiological mechanisms of these comorbidities makes it difficult to isolate the specific mechanisms of SARS-CoV-2 in the rapid deterioration of patients with COVID-19. There is also a discrepancy in adherence of medication which may skew observations and mask potential effects of these drugs, when taken regularly. Large-scale RCTs are needed to explore the interaction of these drugs with target receptors of SARS-CoV-2. Experimental validation of potential target receptors of SAR-CoV-2 and the molecular pathways it employs may reveal additional pre-existing drugs which can be repurposed or modified for use in COVID-19 patients.
